# Psychological health and wellness and the impact of 6 weeks and 3 months supportive text messaging program (Wellness4MDs) among physicians and medical learners in Canada: a longitudinal study

**DOI:** 10.3389/fpubh.2025.1629490

**Published:** 2025-09-04

**Authors:** Samuel Obeng Nkrumah, Reham Shalaby, Belinda Agyapong, Ejemai Eboreime, Charles Kelderhouse, Vincent Israel Opoku Agyapong

**Affiliations:** ^1^Department of Psychiatry, Dalhousie University, Halifax, NS, Canada; ^2^Department of Psychiatry, University of Alberta, Edmonton, AB, Canada

**Keywords:** Wellness4MDs, mental health, text messaging, physician, postgraduate medical trainee, medical students

## Abstract

**Background:**

Physicians and medical learners face high rates of burnout, anxiety, and depression due to the demanding nature of their work. Many are reluctant to seek support because of stigma, time constraints, and limited access to care. Cognitive Behavioral Therapy (CBT)-based supportive SMS messaging offers a promising, scalable alternative.

**Objective:**

This study evaluates the impact of Wellness4MDs, a CBT-based supportive messaging program, on the psychological health and well-being of physicians and medical learners in Canada.

**Methods:**

Participants subscribed to the Wellness4MDs program and received daily supportive SMS messages for 3 months. Standardized self-rated web-based questionnaires assessing depression, anxiety, burnout symptoms were collected at baseline, 6 weeks, and 3 months using the PHQ-9, GAD-7, MBI, and WHO-5. Subscribers’ satisfaction was measured using an online, self-developed questionnaire adapted from tools previously employed to assess similar programs.

**Results:**

A total of 806 subscribers participated, with 226 completing the baseline survey. 66 participants completed surveys at all follow-up points, and 53 completed both baseline and at least one follow-up survey. At the three-month follow-up, there were statistically significant reductions in mean scores for emotional exhaustion (EE) and anxiety symptoms (GAD-7), with reduction from baseline of 16.1% (*t* = 2.86, *p* = 0.01) and 15.5% (*t* = 2.05, *p* = 0.05) with effect sizes of 0.4 and 0.3 respectively, indicating moderate effects. These reductions remained statistically significant when missing data were imputed using the last observation carried forward (LOCF) method. However, no significant changes were observed on the PHQ-9 scale. The overall mean satisfaction score for the Wellness4MDs program was 7.98 (SD = 2.06). Most participants reported that the messages helped them cope with stress (72.7%), anxiety (70.5%), depression (51.1%), and loneliness (42.0%). Additionally, 71.6% felt more connected to a support system, and 78.4% reported improved overall mental well-being.

**Conclusion:**

Wellness4MDs demonstrated effectiveness in reducing emotional exhaustion and anxiety symptoms. Its high user satisfaction, accessibility, and low-cost delivery model make it a promising complement to traditional mental health services for healthcare professionals.

## 1 Introduction

The mental health and psychological well-being of physicians have been extensively documented worldwide, as highlighted in numerous studies (1–3). Evidence shows that physicians experience higher levels of mental distress, burnout, and suicidal ideation compared to other professions (4–6). This psychological distress is particularly prominent during medical school, residency training, and the early stages of a physician’s career (5, 7). The WHO World Mental Health International College Student project, carried out in 19 colleges across eight countries, found that mental disorders among students were both widespread and increasing (8). Research further indicates that medical students experience even higher levels of stress and greater mental health challenges compared to their non-medical peers (9, 10). While significant efforts have been directed toward studying and improving the well-being and quality of care for patients, far less attention has been given to the well-being of physicians and medical learners. Many physicians have overlooked their health as they cope with the pressures of heavy workloads, a rapidly expanding knowledge base, increasing government regulations, malpractice concerns, and the ongoing challenge of balancing personal and professional responsibilities.

Mental health disorders such as depression, anxiety, and burnout remain a significant concern in Canada. A National Physician Survey conducted by the Canadian Medical Association revealed a notable decline in the well-being of physicians across the country (11). The survey reported that only 47% of respondents considered their mental health to be flourishing, a drop from 63% in 2017. Meanwhile, 46% reported moderate mental health, an increase from 33% in 2017, and 7% were classified as having languishing mental health, representing an approximate three-percentage-point increase since 2017. Additionally, more than half (53%) of respondents reported symptoms of burnout, with burnout rates being higher among medical residents (58%) compared to physicians (52%). Furthermore, 1 in 4 respondents experienced anxiety, categorized as either “severe” (10%) or “moderate” (15%), while nearly half (48%) reported experiencing symptoms of depression (11). The Medscape Physician Burnout and Depression Report 2024 found that physician burnout rates reached as high as 49% (12), with elevated levels observed across all medical specialties. Similarly, burnout among medical residents remained high, ranging between 41 and 74% across various specialties (13). Furthermore, 27% of residents reported rarely or never having time for a fulfilling social life, and among these, 68% cited failed relationships as a result (14).

Physicians’ and medical learners’ (postgraduate medical trainees and undergraduate medical students) mental health has continued to decline, particularly during the COVID-19 pandemic, with 60% of Canadian physicians reporting worsening mental well-being (15). Multiple studies have consistently reported high rates of burnout (3, 16, 17), depression (18–20), and anxiety (21–23) among physicians and medical residents. Additionally, mental health and psychological well-being among undergraduate medical trainees (medical students) warrant significant attention due to their serious implications. A meta-analysis revealed that approximately one in three medical students globally (33.8%) experience anxiety, a prevalence notably higher than that of the general population (24). Similarly, a study assessing depression among undergraduate medical students reported overall depression rates ranging between 30.9 and 77.6% (25).

Addressing mental health and psychological well-being among physicians and medical learners is crucial, as many medical professionals typically do not seek psychological support or help. A study conducted among physicians at a university hospital revealed that 3 in 4 distressed physicians had never sought professional help for depression or burnout (26). Additionally, among physicians experiencing burnout or depression, more than half (53%) did not consult a mental health professional and expressed no intention to do so in the future (12). Similarly, only one-third (33.9%) of medical students experiencing burnout sought help for an emotional or mental health issue within the past 12 months (27). Numerous studies have demonstrated that poor psychological health among physicians adversely affects healthcare delivery and physician-related outcomes. These effects include reduced professionalism, increased medical errors, compromised quality and safety of care, and poorer patient outcomes and satisfaction (28–32). Physicians and medical learners often avoid seeking mental health support due to stigma and concerns about their professional reputations. They fear that acknowledging issues like burnout, anxiety, or depression could be viewed as a sign of weakness (33) and negatively impact their careers. Barriers such as limited time, confidentiality concerns, and fear of professional consequences further hinder access to mental health care during residency (34). Additionally, nearly two-thirds (64%) of residents believe there is a stigma surrounding seeking mental health support (14).

Therefore, there is a need for an evidence-based, cost-effective, and technology-enabled mental health service that is accessible regardless of geographic location. Such a service would address the psychological challenges faced by physicians, postgraduate trainees, and medical students while overcoming stigma and barriers to access. In view of this, we introduced the Wellness4MDs intervention, a novel text-based initiative aimed at delivering daily supportive and informative SMS messages—some of which included embedded web links, to offer mental health support and educational resources to physicians and medical learners across Canada. The messages are grounded in cognitive behavioral therapy (CBT) principles and developed collaboratively by psychiatrists, mental health therapists, clinical psychologists, and individuals with lived mental health experience. The primary goal of the Wellness4MDs initiative is to support the mental health and well-being of physicians, postgraduate trainees, and medical students in Canada through daily SMS messages.

In Canada, e-mental health initiatives have shown promise in increasing the use of mental health services (35). Cost-effective and accessible interventions are particularly important, as many individuals with mental health conditions— including physicians and medical learners, often do not seek help due to stigma or geographical barriers (36). Cognitive Behavioral Therapy (CBT), a psychological treatment aimed at changing negative thought patterns (37), has been successfully delivered through text messaging, demonstrating its effectiveness in bridging gaps in mental health care and reducing symptoms of anxiety and depression (38–40).

For example, burnout, comprising emotional exhaustion (EE), depersonalization (DP), and reduced personal accomplishment (PA) (41), can be effectively addressed through Cognitive Behavioral Therapy (CBT)-based interventions. The Wellness4MDs SMS program uses CBT principles to target these dimensions. By promoting stress reappraisal, emotion regulation, and self-compassion, the program helps reduce emotional exhaustion (42). It also aims to counter DP by challenging beliefs that encourage emotional detachment and by reinforcing empathy and professional purpose. CBT strategies such as goal setting and cognitive reframing can improve PA by boosting self-efficacy and motivation (43). These mechanisms offer a strong theoretical basis for using CBT-informed messaging to reduce burnout in healthcare professionals. Additionally, CBT-based text messaging interventions are theoretically grounded in addressing anxiety, depression, and promoting well-being by targeting cognitive distortions and maladaptive coping. Beck’s Cognitive Theory of Anxiety posits that anxiety stems from overestimating threats and underestimating coping abilities (44). CBT-informed messages that challenge catastrophic thinking and promote calming strategies can interrupt this cycle and reduce anxiety. Regarding well-being, daily SMS messages incorporating gratitude, self-kindness, and optimism draw from positive psychology and the Broaden-and-Build Theory, which suggests that cultivating positive emotions broadens psychological resources and builds resilience (45). These CBT strategies have been shown to improve psychological functioning and are increasingly used in digital mental health interventions (46, 47).

Research on supportive SMS text messaging has shown positive outcomes, including improved clinical symptoms and high user satisfaction (39, 48, 49). For instance, the Text4Mood service, SMS text messaging program designed to fill gaps in psychological treatment, reported that the majority of subscribers felt more hopeful about managing their challenges (82%), empowered to handle depression and anxiety (77%), and felt connected to a support system (75%). Additionally, 83% of users indicated improvements in their overall well-being (48). Similarly, Text4Support, another text messaging program, demonstrated significant benefits, including reducing self-harm risk after 6 months of intervention (50) and decreasing symptoms of distress, anxiety, and depression in clinical populations (51). Population-level SMS text messaging programs consistently achieve over 80% user satisfaction, with most subscribers reporting a stronger connection to support systems, improved ability to manage anxiety, depression, and life challenges, and enhanced mental health literacy (48, 49, 52, 53). The key strength of these supportive SMS text messaging programs lies in their feasibility and high perceived acceptability among users. However, there are limitations to these SMS text messaging programs. These text-based interventions may be less effective for more complex or severe mental health conditions, such as major depressive disorder or clinical burnout, which often require personalized, therapist-guided treatment.

The Wellness4MDs program distinguishes itself from the other supportive text messaging initiatives, such as Text4Mood, by specifically targeting physicians and medical learners, addressing the unique mental health challenges faced within the medical profession. The program delivers profession-specific messages that reflect the realities of clinical practice, using language and examples that resonate with healthcare providers. While based on evidence from earlier programs such as Text4Mood and Text4Support, Wellness4MDs is uniquely tailored to the healthcare context, addressing the specific stressors and mental health challenges faced by this population. The program aims to evaluate the probable burnout and mental health symptoms, as well as determine whether the daily supportive SMS messages can reduce the severity of these symptoms and improve overall well-being of physicians and medical learners in Canada.

We hypothesize that: (1) at least a 20% reduction in mean scores for burnout, anxiety, and depressive symptoms on validated scales and a 20% increase in self-reported well-being at 6 weeks and 3 months after receiving the service and (2) at least 80% of subscribers will express satisfaction with the program and perceive the daily supportive SMS text messages as contributing to their overall mental well-being.

## 2 Methods

### 2.1 Study setting and design

This quantitative longitudinal study evaluated the short-term effectiveness of the Wellness4MDs program, a supportive text messaging intervention that delivered daily messages over a three-month period. The study targeted Canadian physicians and medical learners, including postgraduate medical trainees and medical students. Recruitment was conducted nationally through collaborations with the Royal College of Physicians and Surgeons of Canada, faculties of medicine, provincial medical associations, health authorities, and medical licensing bodies. Promotional materials describing the Wellness4MDs program, along with subscription and opt-out instructions, were distributed to potential participants. These materials were shared by partner organizations through member mailing lists and social media platforms such as Facebook, LinkedIn, Instagram, and Twitter (now X).

Inclusion criteria include:

- Members (physicians, postgraduate medical trainees and medical students) of the Royal College of Physicians and Surgeons of Canada, faculties of medicine across Canada, provincial medical associations, provincial health authorities, and provincial medical licensing colleges.
- Ownership of a mobile device capable of receiving text messages.
- Able to read English and French text messages.

Exclusion criteria:

- Cannot read English and French text messages.
- Does not own a mobile phone.

### 2.2 Institutional review board approval

The study received approval from the Health Sciences Research Ethics Board of Dalhousie University (REB#: 2023–6,840) and the Human Ethics Review Board of the University of Alberta (Pro00129541). Informed consent was implied when participants accessed the study information leaflet, completed the survey questions, and submitted their voluntary online responses. Confidentiality and data security protocols were strictly followed, as approved by the ethics boards. Participant privacy was protected through encrypted SMS delivery using the ResilienceNHope platform, secure storage of de-identified data, and access restricted to authorized personnel.

### 2.3 Data collection

Data was collected between December 1, 2023, and September 30, 2024, through the Wellness4MDs program using a web-based survey at baseline (program enrollment), 6 weeks, and 3 months with a REDCap software program (54). The procedure for data collection is fully described in the study protocol (55). Physicians, postgraduate medical trainees (residents), and medical students subscribed to the program by texting “WellMD” for access in English or “BIENMD” for access in French to a designated phone number, which enrolled them to receive daily one-way supportive and informative SMS text messages for 6 months. Examples of an SMS text message with an embedded web link read:

- “Perfectionism can cause anxiety. Remember a perfect work environment does not exist. Try deliberately being imperfect and see what happens. Do your anxious thoughts come true? Remember that there are many possibilities in life. Try not to limit yourself from enjoying all that is possible.” In addition, an embedded web link has been provided (56).
- “Problems are almost never solved in one step. See if you can break the problem into steps. Take the first step today by starting with a manageable situation. Get the support of a friend, family, colleagues, or supervisor.” In addition, an embedded web link has been provided (57).

**Figure 1:**
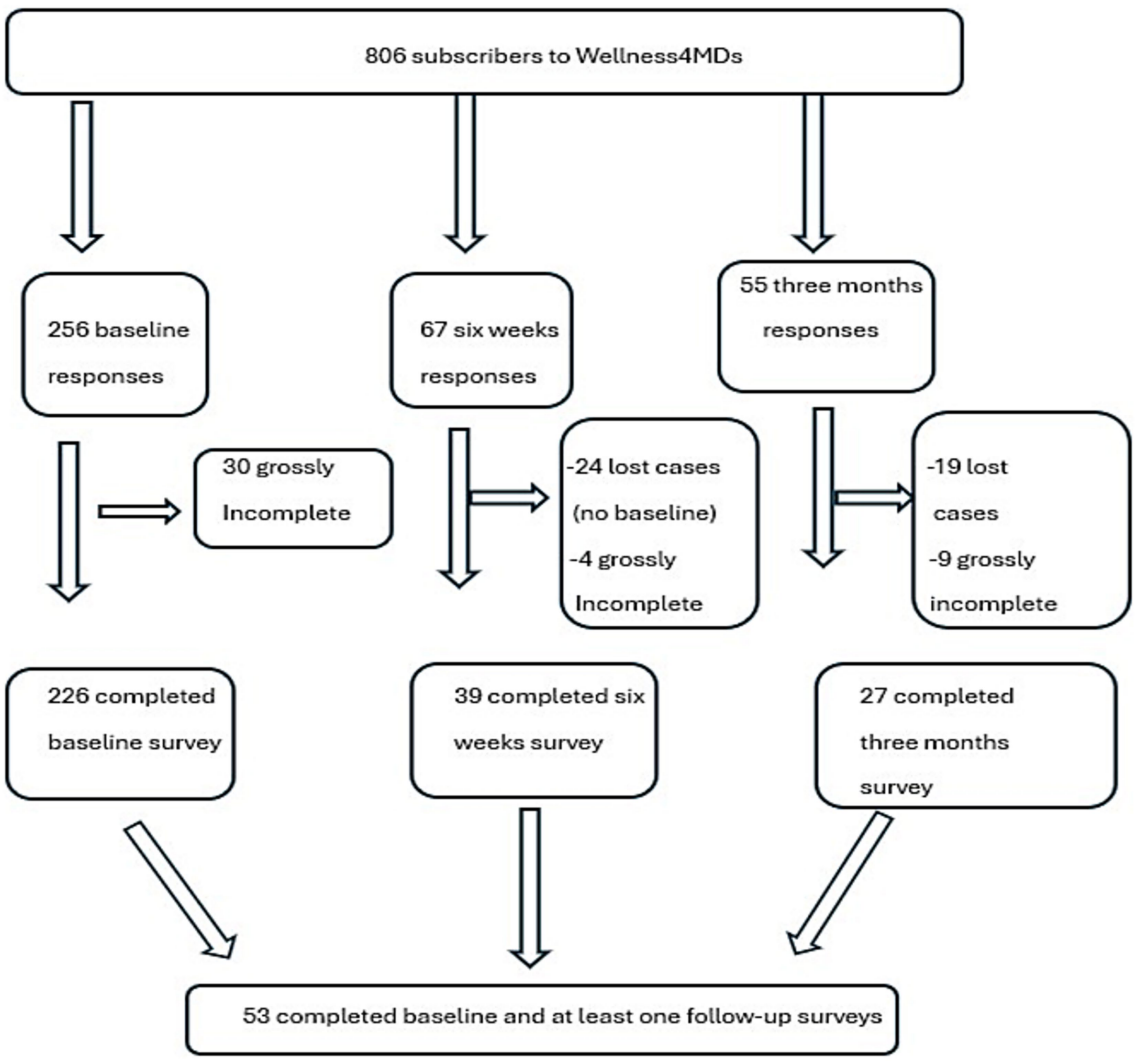
Study flowchart illustrating the total number of subscribers to the Wellness4MDs and the number of surveys collected at each time point.

The SMS text messages were created collaboratively by psychiatrists, mental health therapists, and individuals with lived experience of mental health challenges, and were grounded in cognitive-behavioral therapy (CBT) principles. Participation was voluntary, and participants continued to receive supportive text messages regardless of whether they completed the surveys. Each survey required a maximum of 10 min to complete. Participants could opt out of the program at any time by texting “STOP” to the same number. No incentives were provided for survey completion. The baseline survey collected demographic data and clinical outcome measures. Follow-up survey links were sent via text message 6 weeks and 3 months after enrollment (as illustrated in Figure 1).

In total, 806 participants subscribed to the program. Of these, 256 participants responded at baseline, resulting in a response rate of 32%; however, only 226 completed the baseline survey, while 30 surveys were grossly incomplete. At follow-up, 66 participants provided responses: 39 completed the six-week survey, and 27 completed the three-month survey. A total of 53 participants completed the baseline survey and at least one follow-up survey, while only 14 participants completed the baseline, six-week, and three-month follow-up surveys.

### 2.4 Outcome measures

Primary outcome measure was changes in the mean scores on the Patient Health Questionnaire (PHQ-9), Generalized Anxiety Disorder Scale (GAD-7), the Maslach Burnout Inventory (MBI), and the World Health Organization-Five Well-Being Index (WHO-5) for subscribers from baseline to 6 weeks and 3 months, assessing depression, anxiety, burnout symptoms, and quality of life and mental wellbeing, respectively.

The PHQ-9 is a self-administered scale designed to measure likely depression. It is an effective tool for patients and a validated tool for screening depressive symptoms among the general population (58). It is a nine-item questionnaire measured on a four-point Likert scale from “0” (not at all) to “3” (nearly every day). The PHQ-9 scale categorizes depression scores into none to minimal (0–4 points), mild (5–9 points), moderate (10–14 points), moderately severe (15–19 points), and severe (20–27 points) (59). For analysis purposes, scores were recategorized into two groups: Likely depression (score ≥ 10) and unlikely depression (score < 10). The tool’s reliability and validity have shown good psychometric properties with accepted sensitivity using 10 or above as a cutoff score and high internal consistency (Cronbach *α* = 0.79) (58, 60).

The GAD-7 scale is a self-administered valid tool designed to measure likely anxiety (61, 62). The 7 self-reported items are used to assess the severity of generalized anxiety disorder (GAD) symptoms over the past 2 weeks. Ratings are based on a 4-point Likert scale: not at all sure (0), several days (1), over half the days (2), and nearly every day (3). Scores range from 0 to 21. The scale categorizes anxiety scores as minimal (0–4), mild (5–9), moderate (10–14), and severe anxiety (15–21) (63). A sensitivity of 89% and a specificity of 82% for a cutoff score of 10 (62) are considered. The GAD-7 also showed good test–retest reliability (intraclass correlation 0.83), as well as excellent internal consistency (Cronbach *α* = 0.92) (61, 64). The scores were recategorized into Likely anxiety (score ≥ 10) and unlikely anxiety (score < 10).

The Maslach Burnout Inventory- Human Services Survey for Medical Personnel (MBI-HSS MP) was used to collect data on burnout levels among medical personnel. The MBI-MP contains 22 seven-point items distributed across three dimensions: Emotional Exhaustion (nine items), Personal Accomplishment (eight items), and Depersonalization (five items). The 7-point Likert response scales are defined as follows: 0 (never); 1 (a few times a year or less); 2 (once a month or less); 3 (a few times a month); 4 (once a week); 5 (a few times a week) 6 (every day) (65). Emotional exhaustion scores are categorized as high (≥27), moderate (17–26), or low (0–16). Depersonalization scores are classified as high (≥13), moderate (7–12), or low (0–6). Personal accomplishment scores are interpreted as 0–31 indicating low levels, 32–38 moderate levels, and 39 or above reflecting high levels of personal accomplishment. The reliability of MBI is supported by several studies, where Cronbach *α* ratings of 0.90 for emotional exhaustion, 0.76 for depersonalization, and 0.76 for personal accomplishment (66).

The WHO-5 is a self-reported measure was to assess well-being over time or to compare well-being between groups (67). The scale includes 5 positively worded items with a 6-point Likert scale used to assess participant’s feelings in the last 2 weeks, ranging from 0 (not present) to 5 (constantly present). The raw scores are transformed into a score from 0 (worst thinkable well-being) to 100 (best thinkable well-being) (68). The scale is scored by adding up the values of the five responses, resulting in a raw score ranging from 0 to 25, where 0 indicates the lowest possible quality of life, and 25 indicates the highest. To convert this into a percentage score, the raw score is multiplied by 4, with 0% representing the lowest quality of life and 100% the highest. A total raw score of 13 or less, or a percentage score of 50% or below, signifies low quality of life (QoL) and poor mental well-being and requires further evaluation for depression (67, 69). Conversely, a raw score above 13 or a percentage score exceeding 50% reflects a high quality of life (QoL). The WHO-5 demonstrated satisfactory internal consistency reliability and concurrent validity with other scales (67, 68).

### 2.5 Sample size considerations

With a projection that the effect size for the reduction in mean scores of GAD-7 scale, (PHQ-9, and MBI) scores at 3 months from baseline would be 0.1, a population variance of 1 for each scale mean score, a 2-sided significance level *α* = 0.05, and a power of 90% (*β* = 0.1), using a web-based script (70), we estimated that the sample size needed to assess the effects of the daily supportive SMS text messages on the outcome variables would be 1,053. Given the nature of the Wellness4MDs program—a low-intensity, low-risk supportive text messaging intervention designed to promote mental well-being, a small effect size can still have a meaningful public health impact, especially when applied to high-risk groups like medical professionals. For a similar intervention, Text4Hope, effect sizes ranged from 0.1 to 0.5 in the general population with respect to changes in mean scores for resilience, wellbeing, anxiety, and depression (71). Based on this, we used a conservative effect size estimate of 0.1 in our sample size calculation to evaluate the impact of the Wellness4MDs program.

### 2.6 Statistical analysis

SPSS for windows, version 25(IBM Corporation, Armonk, NY, United States) (72) was used to analyze the data for this study. Participants’ roles, (physician, postgraduate medical trainee, and medical student) were plotted against all independent variables. The Chi-square/Fisher’s exact test was employed to determine the relationship between each of the sociodemographic as categorical variables. Also, to determine the relationship between the clinical variables (continuous variables) among the study participants, a one-way analysis of variance (ANOVA) was conducted. Welch’s ANOVA was employed where appropriate to account for violations of the homogeneity of variance assumption. Percentages and numbers were used to report the descriptive characteristics, with a significance level of *p* ≤ 0.05 used to determine statistical significance for all analyses. Similarly, to examine the differences in the mean scores of the clinical scales at baseline and those who completed follow-up surveys (6 weeks and 3 months), a paired sample 2-tailed *t*-test was performed. Paired sample *t*-tests were used to assess changes in continuous variables between baseline and follow-up. Before applying the paired sample *t*-tests, the normality assumption was assessed using the Shapiro–Wilk test, suitable for smaller sample sizes. We also conducted Levene’s test to check for homogeneity of variances. To handle missing data, we utilized imputation techniques, focusing on the Last Observation Carried Forward (LOCF) method. This approach replaces missing values with the most recent recorded observations for the same individual. For example, participants with missing responses at 3 months had their missing data imputed, employing the last observations carried forward, precisely their responses at 6-week. By applying LOCF, we included all participants who completed the follow-up surveys and had baseline data in the analysis.

## 3 Results

Table 1 presents the sociodemographic characteristics of participants enrolled in the Wellness4MDs program against participants’ role. The age distribution varied across professional roles, with vast majority of medical students aged ≤30 years (88.9%), while nearly half of postgraduate trainees fell into the same age group (47.4%), with a notable proportion being older. In contrast, most physicians were aged between 41 and 50 years (39.5%) or ≥51 years (34.2%). Gender distribution was relatively consistent across all groups, with the majority of participants identifying as female (*n* = 186, 82.3%). Ethnic composition showed minor variations, though Caucasians comprised the majority in each group (*n* = 162, 71.7%). Relationship status differed significantly: while most participants were in a common-law relationship, partnered, or married (*n* = 165, 73.0%), medical students had a higher proportion of single individuals (*n* = 10, 27.8%) compared to physicians and trainees. Family practice was the most commonly reported specialty (*n* = 60, 32.1%). Geographically, the largest number of participants resided in Ontario (*n* = 65, 28.8%), followed by Alberta and Nova Scotia. Housing status also varied by role, with the majority of physicians (*n* = 137, 90.1%) reporting home ownership.

**Table 1:** Distribution of socio-demographics among the study participants.

Variables	Role				Chi2/Fishers Exact* test values	*p*-values
	Physician *n* (%)	Post graduate Medical trainee *n* (%)	Medical student *n* (%)	Total *n* (%)		
Age (*N* = 226)						
≤ 30 years	3 (2)	18 (47.4)	32 (88.9)	53 (23.5)	149.6	<0.001
31–40 years	37 (24.3)	13 (34.2)	4 (11.1)	54 (23.9)		
41–50 years	60 (39.5)	5 (13.2)	0 (0)	65 (28.8)		
≥51 years	52 (34.2)	2 (5.3)	0 (0)	54 (23.9)		
Gender (*N* = 226)						
Male	27 (17.8)	7 (18.4)	6 (16.7)	40 (17.7)	0.04	1.0
Female	125 (82.2)	31 (81.6)	30 (83.3)	186 (82.3)		
Ethnicity (*N* = 226)						
Caucasian	116 (76.3)	20 (52.6)	26 (72.2)	162 (71.7)	14.08*	0.02
Asian	21 (13.8)	12 (31.6)	7 (19.4)	40 (17.7)		
African/Latino	3 (2.0)	4 (10.5)	2 (5.6)	9 (4.0)		
Others	12 (7.9)	2 (5.3)	1 (2.8)	15 (6.6)		
Relationship status (*N* = 226)						
Common-law, partnered or MARRIED	110 (72.4)	29 (76.3)	26 (72.2)	165 (73.0)	16.70*	0.005
Single	15 (9.9)	6 (15.8)	10 (27.8)	31 (13.7)		
Separated or divorced	25 (16.4)	2 (5.3)	0 (0.0)	27 (11.9)		
Widowed	2 (1.3)	1 (2.6)	0 (0.0)	3 (1.3)		
If you are a physician or postgraduate medical trainee, what is your speciality? (*N* = 187)						
Family practice	51 (33.6)	9 (25.7)	N/A	60 (32.1)	6.65*	0.42
Internal Medicine and related specialities (e.g., neurology, cardiology, respiratory medicine etc.)	21(13.8)	3 (8.6)	N/A	24 (12.8)		
Psychiatry	18 (11.8)	4 (11.4)	N/A	22 (11.8)		
Surgical speciality	17 (11.2)	2 (5.7)	N/A	19 (10.2)		
Anesthesia	8 (5.3)	2 (5.7)	N/A	10 (5.3)		
Radiology and intervention medicine	4 (2.6)	1 (2.9)	N/A	5 (2.7)		
Pathology and laboratory medicine	3 (2.0)	0 (0.0)	N/A	3 (1.6)		
Other	30 (19.7)	14 (40.0)	N/A	44 (23.5)		
If you are a medical student or post-graduate medical trainee, which year of the program are you in? (*N* = 69)						
First year	N/A	13 (37.1)	5 (14.7)	18 (26.1)	6.90	0.08
Second year	N/A	8 (22.9)	10 (29.4)	18 (26.1)		
Third year	N/A	5 (14.3)	12 (35.3)	17 (24.6)		
Fourth year	N/A	9 (25.7)	7 (20.6)	16 (23.2)		
Where do you currently reside (*N* = 226)						
Alberta	35 (23.0)	5 (13.2)	6 (16.7)	46 (20.4)	*	
British Columbia	13 (8.6)	6 (15.8)	7 (19.4)	26 (11.5)		
Saskatchewan	6 (3.9)	0 (0)	0 (0)	6 (2.7)		
New brunswick	11 (7.2)	1 (2.6)	0 (0)	12 (5.3)		
Manitoba	3 (2.0)	3 (7.9)	1 (2.8)	7 (3.1)		
Quebec	5 (3.3)	0 (0)	0 (0)	5 (2.2)		
Nova Scotia	23 (15.1)	5 (13.2)	2 (5.6)	30 (13.3)		
Ontario	35 (23.0)	16 (42.1)	14 (38.9)	65 (28.8)		
New foundland and labrador	6 (3.9)	2 (5.3)	1 (2.8)	9 (4.0)		
Nunavut	12 (7.9)	0 (0)	5 (13.9)	17 (7.5)		
Yukon	3 (2.0)	0 (0)	0 (0)	3 (1.3)		
Housing status (*N* = 226)						
Own Home	137 (90.1)	16 (42.1)	10 (27.8)	163 (72.1)	79.10*	0.001
Rented accommodation	14 (9.2)	19 (50.0)	18 (50.0)	51 (22.6)		
Live with family or friends	1 (0.7)	3 (7.9)	8 (22.2)	12 (5.3)		

Table 2 presents the results of one-way analyses of variance (ANOVA) examining differences in clinical characteristics among physicians, postgraduate medical trainees, and medical students at baseline. The clinical measures assessed included the WHO-5, GAD-7, PHQ-9, and MBI. Medical students reported the highest average well-being (*M* = 13.03, SD = 5.12), followed by physicians (*M* = 12.03, SD = 4.94) and trainees (*M* = 10.83, SD = 4.68), although these differences were not statistically significant. In contrast, for GAD-7 scores, medical students also reported the highest mean anxiety score (*M* = 8.85, SD = 5.12), followed by physicians (*M* = 7.66, SD = 5.40) and trainees (*M* = 7.46, SD = 5.22), indicating greater anxiety symptoms among students. However, these differences were not statistically significant. Similarly, depression scores were highest among trainees (*M* = 8.67, SD = 6.37) and lowest among medical students (*M* = 7.35, SD = 5.26), but these differences were also not statistically significant. The most notable differences emerged in emotional exhaustion, a core dimension of burnout. Physicians reported the highest levels of emotional exhaustion (*M* = 31.69, SD = 12.58), followed by trainees (*M* = 30.10, SD = 12.48), and medical students (*M* = 24.83, SD = 9.81). Welch’s ANOVA indicated these differences were statistically significant [*F* (2, 68) = 6.06, *p* = 0.004]. Depersonalization scores followed a similar pattern, with higher mean scores among physicians (*M* = 9.55, SD = 6.69) and trainees (*M* = 9.43, SD = 6.63) compared to medical students (*M* = 7.34, SD = 5.24), although these differences were not statistically significant. Finally, personal accomplishment scores were highest among physicians (*M* = 37.30, SD = 6.67) and lowest among medical students (*M* = 34.23, SD = 7.36), but no significant group differences were observed.

**Table 2:** One-way ANOVA distribution of clinical characteristics at baseline among the study participants.

Clinical conditions severity, Mean (SD)					
Variables	Role	*N*	Mean (SD)	ANOVA Values (df), *F* values.	*p*-value
WHO-5	Physician	139	12.03 (4.94)	(2, 205), 1.74	0.18
	Post Graduate Medical Trainee	35	10.83 (4.68)		
	Medical Student	34	13.03 (5.12)		
	Total	208	11.99 (4.94)		
GAD-7	Physician	138	7.66 (5.40)	(2, 204), 0.78	0.46
	Post Graduate Medical Trainee	35	7.46 (5.22)		
	Medical Student	34	8.85 (5.12)		
	Total	207	7.82 (5.32)		
PHQ-9	Physician	138	7.40 (5.58)	(2, 204), 0.77	0.47
	Post Graduate Medical Trainee	35	8.69 (6.37)		
	Medical Student	34	7.35 (5.26)		
	Total	207	7.60 (5.66)		
Emotional exhaustion (EE)	Physician	144	31.69 (12.58)	* (2, 68),6.06	0.004
	Post Graduate Medical Trainee	35	30.10 (12.48)		
	Medical student	35	24.83 (9.81)		
	Total	214	30.30 (12.36)		
Depersonalization (DP)	Physician	144	9.55 (6.69)	(2, 211), 1.67	0.20
	Post graduate medical trainee	35	9.43 (6.63)		
	Medical student	35	7.34 (5.24)		
	Total	214	9.17 (6.49)		
Personal accomplishment (PA)	Physician	144	37.30 (6.67)	(2, 211), 2.80	0.06
	Post graduate medical trainee	35	36.49 (7.14)		
	Medical student	35	34.23 (7.36)		
	Total	214	36.65 (6.92)		

*Welch ANOVA.

Table 3 illustrates *post hoc* comparisons exploring pairwise differences in emotional exhaustion (EE) among physicians, postgraduate medical trainees, and medical students. The analysis reveals that physicians reported significantly higher emotional exhaustion than medical students, with a mean difference of 6.86 (SE = 2.29, *p* = 0.009), and a 95% confidence interval ranging from 1.45 to 12.27.

**Table 3:** Post hoc analysis of emotional exhaustion (EE) by professional role.

Variable	(I) What is your role?	(J) What is your role?	Mean difference	Standard error	*p*-value	95% Confidence Interval	
						Lower bound	Upper bound
Emotional Exhaustion (EE)	Physician	Post graduate medical trainee	1.63	2.29	0.760	−3.78	7.04
		Medical student	6.86*	2.29	0.009	1.45	12.27
	Post graduate Medical trainee	Physician	−1.63	2.29	0.760	−7.04	3.78
		Medical student	5.23	2.91	0.173	−1.63	12.09
	Medical students	Physician	−6.86*	2.29	0.009	−12.27	−1.45
		Post graduate medical trainee	−5.23	2.91	0.173	−12.09	1.63

*Significance at *p* < 0.05.

We conducted a paired sample two-tailed t-test to assess differences in the mean scores of clinical scales between baseline and follow-up time points (6 weeks and 3 months), as shown in Table 4. The WHO-5 total score showed significant improvements, with a 10.44% increase from baseline to the six-week follow-up (*t* = −2.13, *p* = 0.04), with a low effect size (Cohen’s d = 0.28). When combining follow-up data using the last observation carried forward (LOCF) method, WHO-5 scores demonstrated a significant overall increase with a 9.40% change from baseline (*t* = −2.06, *p* = 0.045), with a low effect size (Cohen’s d = 0.2), indicating improved well-being over time. Anxiety symptoms, measured by the GAD-7 scale, consistently decreased across follow-up points. Although the reduction at 6 weeks was not statistically significant (*p* = 0.16), the decrease at 3 months showed a stronger trend toward significance with a 15.5% change in mean score (*t* = −2.05, *p* = 0.05), with a low effect size (Cohen’s d = 0.3). The combined LOCF analysis showed a significant reduction in anxiety symptoms with an overall improvement of approximately 12.6% change in mean score (*t* = 2.16, *p* = 0.04), with a low effect size (Cohen’s d = 0.2).

**Table 4:** Changes in mental health measures from baseline to follow-up time points.

Measures	*N*	Mean Scores (SD)		Mean difference (95% CI)	% Change from baseline	*p* value	*t*-test	Effect size (Cohen’s *d*)
		Baseline	6 weeks					
WHO-5 total score	39	12.26 (4.10)	13.54 (4.99)	−1.28 (−2.50, −0.06)	10.44	0.04	−2.13	0.28
GAD-7 total score	39	6.36 (3.91)	5.64 (4.34)	0.72 (−0.30, 1.74)	11.30	0.16	1.42	017
PHQ-9 total score	39	6.64 (4.48)	6.02 (4.73)	0.62 (−0.45, 1.68)	9.20	0.25	1.17	0.13
Emotional Exhaustion (EE) total score	40	32.10 (12.99)	29.15 (13.56)	2.95 (0.13, 5.77)	9.20	0.04	2.12	0.22
Depersonalization (DP) total score	40	8.92 (7.01)	9.05 (7.45)	−0.13 (−1.55, 1.30)	1.46	0.86	−0.18	0.02
Personal Accomplishment (PA) total score	40	36.15 (6.48)	36.87 (6.79)	−0.73 (−2.45, 0.99)	2.00	0.40	−0.85	0.11
		Baseline	3 months					
WHO-5 total score	26	11.27 (5.17)	12.81 (5.51)	−1.54 (−3.26, 0.18)	13.7	0.08	−1.84	0.3
GAD-7 total score	26	8.38 (4.42)	7.08 (4.78)	1.30 (−0.004, 2.62)	15.5	0.05	2.05	0.3
PHQ-9 total score	26	8.23 (6.19)	7.65 (5.60)	0.58 (−0.94, 2.09)	7.0	0.44	0.78	0.1
Emotional Exhaustion (EE) total score	27	30.63 (11.12)	25.70 (11.04)	4.93 (1.39, 8.46)	16.1	0.01	2.86	0.4
Depersonalization (DP) total score	27	7.70 (5.94)	7.18 (5.80)	0.52 (−0.76, 1.80)	6.8	0.41	0.84	0.1
Personal Accomplishment (PA) total score	27	37.15 (7.52)	36.59 (7.54)	0.56 (−1.08, 2.19)	1.5	0.49	0.70	0.1
		Baseline	All data (6 weeks or 3 months)-LOCF					
WHO-5 total score	52	11.94 (4.52)	13.06 (5.29)	−1.12 (−2.20, −0.03)	9.40	0.045	−2.06	0.2
GAD-7 total score	52	7.13 (4.46)	6.23 (4.77)	0.90 (0.07, 1.74)	12.6	0.04	2.16	0.2
PHQ-9 total score	52	7.40 (5.52)	6.75 (5.31)	0.65 (−0.36, 1.66)	8.8	0.20	1.30	0.1
Emotional Exhaustion (EE) total score	53	31.77 (12.10)	28.30 (13.06)	3.47 (1.11, 5.83)	10.9	0.01	2.95	0.3
Depersonalization (DP) total score	53	8.40 (6.48)	8.51 (6.93)	−0.11 (−1.16, 0.93)	1.3	0.83	−0.22	0.02
Personal Accomplishment (PA) total score	53	36.75 (6.56)	36.64 (7.11)	0.11 (−1.22, 1.45)	0.3	0.87	0.17	0.02

Depression symptoms, as measured by the PHQ-9, showed slight reductions at both the six-week and three-month follow-up points; however, these changes were not statistically significant (*t* = 1.17, *p* = 0.25 at 6 weeks; *t* = 0.78, *p* = 0.44 at 3 months). Emotional exhaustion (EE) scores significantly decreased over time, with a 9.2% reduction in mean score from baseline to 6 weeks (*t* = 2.12, *p* = 0.04), with a low effect size (Cohen’s d = 0.22) and a 16.1% reduction at 3 months (*t* = 2.86, *p* = 0.01), with a low effect size (Cohen’s d = 0.4). The LOCF analysis also show a significant decrease with a 10.9% change in mean score (*t* = 2.95, *p* = 0.01), with a low effect size (Cohen’s d = 0.3). Depersonalization (DP) scores did not show any statistically significant differences (*p* > 0.40). Similarly, personal accomplishment (PA) scores remained stable, with no significant changes observed from baseline to the follow-up time points (*p* > 0.40).

### 3.1 Perceived impact of the Wellness4MDs program

**Figure 2:**
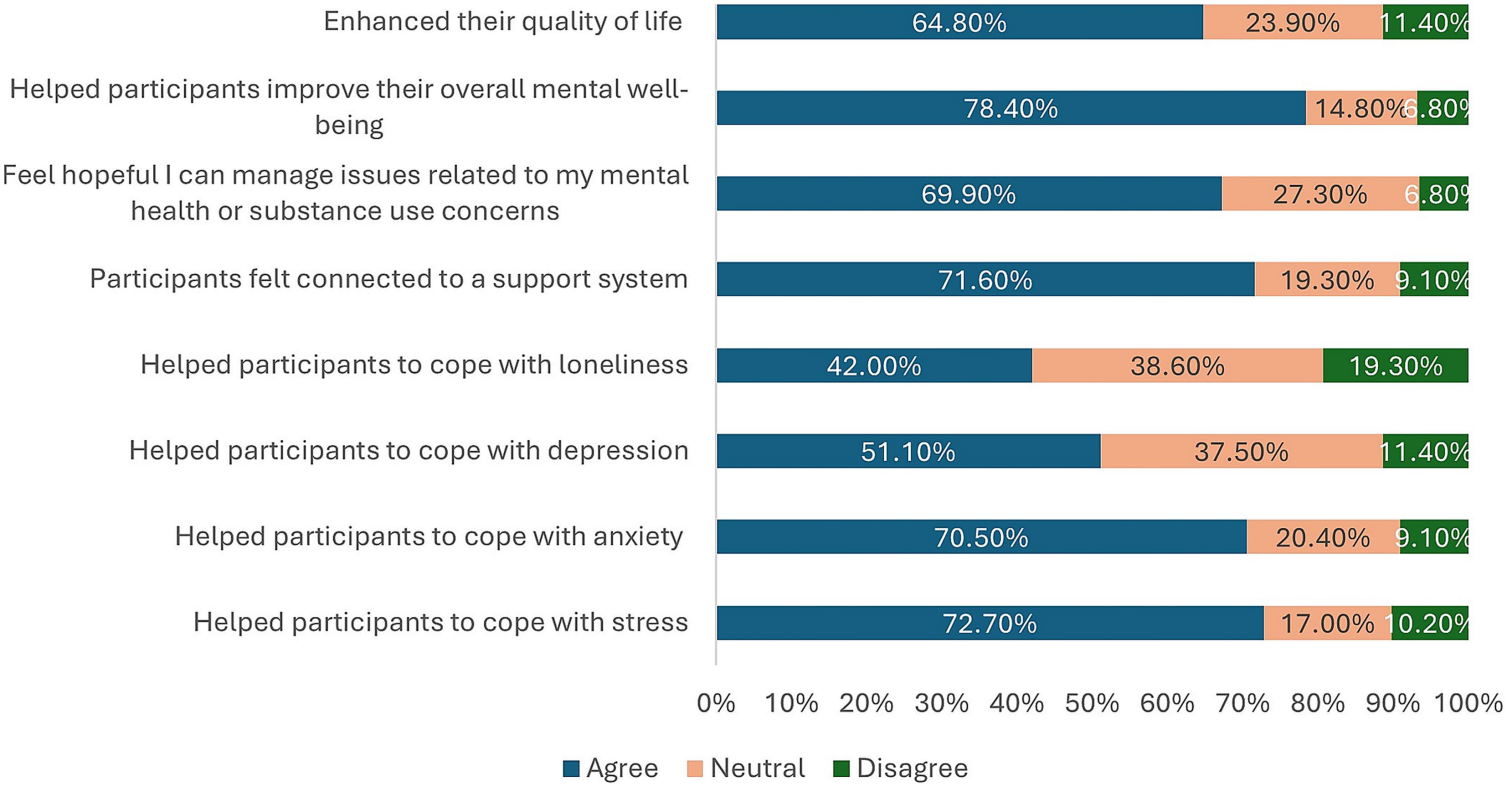
Perceived impact of daily messages post-intervention by participants.

A total of 88 survey responses were received from participants after completing the Wellness4MDs intervention. Table 5 summarizes participants’ perceptions of the impact of the daily supportive text messages. Overall, 64 participants (72.7%) reported that the Wellness4MDs messages helped them cope with stress, while 62 participants (70.5%) indicated that the messages assisted them in coping with anxiety. Additionally, 45 participants (51.1%) reported that the messages supported them in managing symptoms of depression, and 37 participants (42.0%) stated that the messages helped them cope with feelings of loneliness. Beyond symptom-specific support, 63 participants (71.6%) agreed that the Wellness4MDs messages contributed to a sense of connection to a support system. Similarly, 58 participants (69.9%) indicated that the messages fostered hopefulness regarding the management of mental health or substance use concerns. Furthermore, a majority of respondents (69 participants, 78.4%) endorsed that the Wellness4MDs messages contributed to improvements in their overall mental well-being. Lastly, 57 participants (64.8%) agreed that the supportive text messages enhanced their overall quality of life (See Figure 2).

**Table 5:** Perceived impact of daily messages post-intervention by participants.

Perceived impact of wellness4MDs	All participants, *n* (%) *N = 88*
Helped participants to cope with stress	
Agree	64 (72.7)
Neutral	15 (17.0)
Disagree	9 (10.2)
Helped participants to cope with anxiety	
Agree	62 (70.5)
Neutral	18 (20.5)
Disagree	8 (9.1)
Helped participants to cope with depression	
Agree	45 (51.1)
Neutral	33 (37.5)
Disagree	10 (11.4)
Helped participants to cope with loneliness	
Agree	37 (42.0)
Neutral	34 (38.6)
Disagree	17 (19.3)
Participants felt connected to a support system	
Agree	63 (71.6)
Neutral	17 (19.3)
Disagree	8 (9.1)
Feel hopeful I can manage issues related to my mental health or substance use concerns	
Agree	58 (69.9)
Neutral	24 (27.3)
Disagree	6 (6.8)
Helped participants improve their overall mental well-being	
Agree	69 (78.4)
Neutral	13 (14.8)
Disagree	6 (6.8)
Enhanced their quality of life	
Agree	57 (64.8)
Neutral	21 (23.9)
Disagree	10 (11.4)

### 3.2 Participant feedback on the Wellness4MDs program

**Figure 3:**
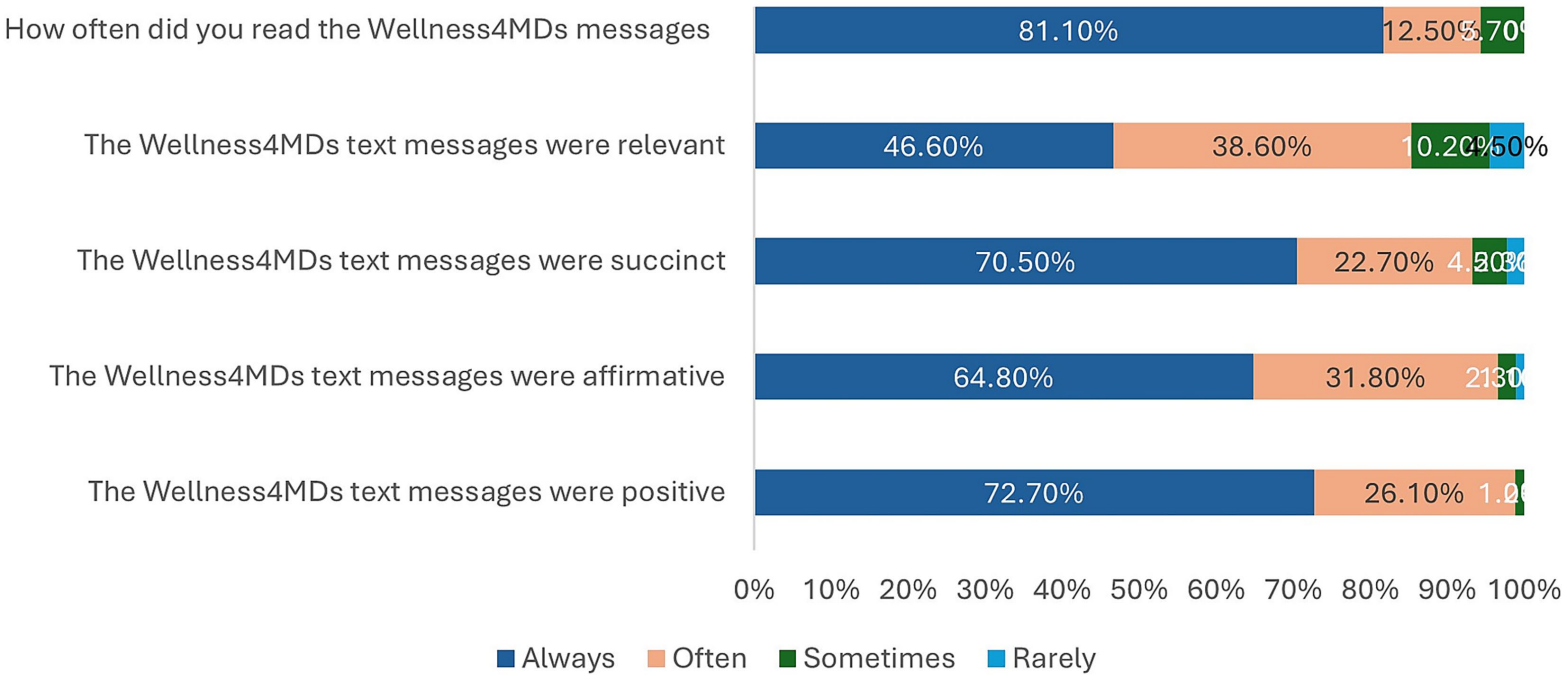
Participants’ feedback after the intervention.

Table 6 presents participants’ feedback regarding the Wellness4MDs supportive text messaging program. Of the 88 respondents, 87 (98.8%) reported that the messages were perceived as “always” or “often” positive, and 85 participants (96.6%) indicated that the messages were “always” or “often” affirming. Similarly, 82 participants (93.2%) rated the messages as “always” or “often” succinct, while 75 (85.2%) found them to be “always” or “often” relevant to their experiences. In terms of engagement, the majority of participants (83, 93.6%) reported that they “always” or “often” read the Wellness4MDs messages (See Figure 3). Additionally, 65 respondents (73.9%) indicated that they not only read the messages but also took time to reflect on them. However, only a small proportion (5 participants, 5.7%) reported reading the messages and subsequently engaging in a positive or beneficial action as a result. Regarding repeated engagement, over half of the participants (46, 52.3%) stated that they “often” or “sometimes” returned to re-read the messages. 74 participants (84.1%) expressed satisfaction with the frequency of the Wellness4MDs text messages. When asked about preferred frequency, the majority (53 participants, 60.2%) indicated a preference for receiving supportive messages once daily, while 23.9% of the respondents preferred to receive the messages Once every other day.

**Table 6:** Participants’ feedback after the intervention.

Feedback	All participants, *n* (%) *N = 88*
The Wellness4MDs text messages were positive	
Always	64 (72.7)
Often	23 (26.1)
Sometimes	1 (1.2)
The Wellness4MDs text messages were affirmative	
Always	57 (64.8)
Often	28 (31.8)
Sometimes	2 (2.3)
Rarely	1 (1.1)
The Wellness4MDs text messages were succinct	
Always	62 (70.5)
Often	20 (22.7)
Sometimes	4 (4.5)
Rarely	2 (2.3)
The Wellness4MDs text messages were relevant	
Always	41(46.6)
Often	34 (38.6)
Sometimes	9 (10.2)
Rarely	4 (4.5)
How often did you read the Wellness4MDs messages	
Always	72 (81.1)
Often	11 (12.5)
Sometimes	5 (5.7)
Most of the time after receiving the Wellness4MDs messages:	
I read the text and took no action	14 (15.9)
I read the text and took time to reflect on the message	65 (73.9)
I read the text and took a positive or beneficial action	5 (5.7)
I read the text and took a negative or harmful action	2 (2.3)
I did not read the text	2 (2.3)
Did you return to read the Wellness4MDs text messages	
Always	4 (4.5)
Often	8 (9.1)
Sometimes	38 (43.2)
Rarely	32 (36.4)
Never	6 (6.8)
How satisfied were you with the frequency of the Wellness4MDs messages?	
Satisfied	74 (84.1)
Mixed	10 (11.4)
Dissatisfied	4 (4.5)
How often would you prefer to receive supportive messages?	
Twice daily	2 (2.3)
Once daily	53 (60.2)
Once every other day	21 (23.9)
Once weekly	12 (13.6)

### 3.3 Participant overall satisfaction with the Wellness4MDs program

Table 7 summarizes participants’ overall satisfaction ratings with the Wellness4MDs program. Of the total respondents, 86 participants completed the satisfaction survey item, which asked them to rate their satisfaction on a scale from 1 (“not at all satisfied”) to 10 (“very satisfied”). The mean satisfaction score was 7.98 (SD = 2.06).

**Table 7:** How participants rated the overall satisfaction with the Wellness4MDs.

Variable	*N*	Mean	Standard deviation	Standard error mean
Using any number from 1 (not at all satisfied) to 10 (very satisfied), how would you rate your overall satisfaction with the Wellness4MDs?	86	7.98	2.06	0.22

## 4 Discussion

This study evaluated the psychological health and wellbeing of Physicians and medical learners, including postgraduate medical trainees and medical students using Wellness4MDs program, a novel CBT-based supportive text messaging program. Overall, the results indicate significant reduction in mean scores in emotional exhaustion (EE), anxiety (GAD-7), and well-being (WHO-5), while depression (PHQ-9), depersonalization (DP), and personal accomplishment (PA) showed no significant changes. The Wellness4MDs program achieved high satisfaction among participants.

### 4.1 Effectiveness of the intervention on clinical outcomes

The results demonstrated a significant reduction in emotional exhaustion (EE), a principal component of burnout. Participants exhibited a 9.2% decrease in EE at 6 weeks (*p* = 0.04), a 16.1% decrease at 3 months (*p* = 0.01), and a 10.9% overall reduction in mean scores (*p* = 0.01) across follow-up periods using the last observation carried forward (LOCF) method, with a small-to-moderate effect size (Cohen’s d = 0.3). The findings suggest that the Wellness4MDs intervention was effective in mitigating emotional exhaustion consistently across the study period. Emotional exhaustion is a critical predictor of physician burnout and has been associated with reduced professional satisfaction, impaired work-life balance, decreased quality of patient care, lower patient satisfaction, and heightened risks to patient safety (7). These findings align with prior research demonstrating that structured wellness interventions can reduce burnout among healthcare professionals (73). Moreover, the results are consistent with those reported by L’Engle et al. (74), who found improvements in burnout following the implementation of a digital wellness program. In contrast, depersonalization (DP) and personal accomplishment (PA) scores demonstrated minimal changes from baseline to follow-up, with no statistically significant improvements observed. Depersonalization, characterized by emotional disengagement from patients and work, remains a central dimension of burnout (41, 75). The absence of a significant reduction in DP suggests that while the Wellness4MDs intervention effectively addressed emotional exhaustion, it may not have sufficiently targeted the broader attitudinal and behavioral elements of burnout. This observation is consistent with a systematic review by Panagioti et al. (76), which concluded that while individual-focused interventions, such as mindfulness and stress management programs, can reduce emotional exhaustion, meaningful improvements in depersonalization and professional efficacy typically require broader organizational interventions, such as workload adjustments and enhanced institutional support.

Following 3 months of participation in the Wellness4MDs program, participants also experienced a significant reduction in anxiety symptoms, as measured by the GAD-7 scale. The three-month follow-up demonstrated a 12.6% reduction in anxiety symptoms (*p* = 0.04), with an associated effect size of 0.2. This finding is consistent with previous research demonstrating reductions in anxiety following supportive text messaging interventions during the COVID-19 pandemic (38) and aligns with broader literature supporting the efficacy of digital health interventions, including mindfulness- and CBT-based programs, in reducing anxiety among healthcare professionals (77). Given the high prevalence of anxiety in this population, these findings highlight the potential value of integrating digital mental health supports into healthcare settings to promote physician and medical learners’ mental health and resilience. Furthermore, the reductions in emotional exhaustion and anxiety may be clinically meaningful for physicians and medical learners, potentially leading to improved coping, a lower risk of medical errors, and enhanced patient interactions.

The study also revealed significant improvements in participants’ psychological well-being. WHO-5 scores showed a 10.44% increase at the six-week follow-up (*p* = 0.04). Moreover, when combining follow-up data using the LOCF method, a 9.40% mean improvement was confirmed (*p* = 0.045), with an effect size of 0.2, indicating sustained enhancement of well-being over the study period. These findings are consistent with existing evidence demonstrating that digital mental health interventions can improve psychological well-being among workers (48, 78) and enhance occupational functioning. Although there was a slight reduction in depression symptoms, as measured by the PHQ-9, across follow-up periods, the observed changes were not statistically significant. This finding contrasts with previous studies that have demonstrated significant reductions in depressive symptoms following digital interventions (79–81). Multiple studies utilizing text-based or internet-delivered CBT interventions have reported improvements in depression outcomes (40, 82, 83). The lack of statistically significant improvement in depression in the present study may be attributable to the relatively short intervention duration or the nature of the intervention, which, while supportive, may not provide the intensity of therapeutic engagement required to treat clinical depression effectively.

The findings of our study suggest that the Wellness4MDs program was effective in improving emotional exhaustion, reducing anxiety symptoms, and enhancing overall well-being among physicians and medical learners. However, the intervention had a limited impact on depersonalization, personal accomplishment, and depression. These differential effects offer important theoretical and practical insights into how text-based, CBT-informed interventions impact different dimensions of mental health and burnout. The improvement in emotional exhaustion and anxiety aligns with cognitive-behavioral models of stress and emotional regulation. Emotional exhaustion (EE) is closely linked to perceived stress and maladaptive appraisal of workplace demands (42). Similarly, anxiety is often fueled by catastrophic thinking and heightened threat perception (44). Wellness4MDs messages, grounded in CBT principles, likely targeted these cognitive distortions directly by offering supportive reframing, reminders of coping abilities, and stress management strategies which helped reduce perceived emotional strain and hyperarousal. In contrast, depression and depersonalization represent deeper, more entrenched cognitive and emotional patterns. Depression often involves persistent negative self-schemas and behavioral withdrawal, while depersonalization reflects emotional disengagement and cynicism developed over time (75). These symptoms may require more intensive, personalized, or therapist-guided interventions to achieve meaningful improvement. Brief generalized text messages may not have been sufficient to modify these more ingrained patterns, especially in a short three-month intervention window.

### 4.2 Participant satisfaction and perceived impact of the Wellness4MDs program

This study evaluated participants’ satisfaction with the Wellness4MDs program following a three-month period of receiving supportive text messages. Findings indicated a high level of overall satisfaction, with a mean satisfaction score of 7.98 (SD = 2.06). This result is consistent with previous studies that have reported high levels of participant satisfaction with supportive text messaging interventions aimed at improving mental health outcomes (39, 52, 84, 85). For example, a similar SMS-based intervention implemented during the COVID-19 pandemic reported a mean satisfaction score of 8.55 (52), comparable to the present findings. Although slightly lower, the satisfaction score for Wellness4MDs aligned with the 95% satisfaction rate reported in the Text4Mood program evaluation (48). Beyond overall satisfaction, the majority of participants reported positive impacts on mental health domains following the intervention. Specifically, 72.7% of participants indicated that the text messages helped them cope with stress, 70.5% with anxiety, 51.1% with depression, and 42.0% with feelings of loneliness. Additionally, 71.6% reported feeling more connected to a support system post-intervention. Participants also perceived broader benefits to their mental health and quality of life. Approximately 69.9% of respondents reported feeling more hopeful about managing mental health or substance use concerns, 78.4% indicated improvements in their overall mental well-being, and 64.8% reported an enhancement in their overall quality of life. These outcomes are largely consistent with findings from similar digital mental health interventions. For instance, the Text4Hope program reported that after 6 weeks of supportive text messaging, 76% of participants indicated an improved ability to cope with anxiety, 56% with depression, and 49% with loneliness. Notably, a slightly higher proportion of Wellness4MDs participants (78.4%) reported improvements in overall mental well-being compared to the 75.6% improvement rate observed in the Text4Hope program (52). Conversely, higher levels of improvement in mental well-being were observed in evaluations of the Text4Mood (48). These findings suggest that the Wellness4MDs program was well-received and effective in enhancing psychological coping, well-being, and quality of life among participating physicians, postgraduate medical trainees, and medical students. The results further reinforce the potential of supportive text messaging as a scalable and accessible mental health intervention.

### 4.3 Participant perceptions of Wellness4MDs message content and delivery frequency

Consistent with findings from prior research, the majority of Wellness4MDs subscribers reported highly favorable perceptions of the supportive text messages received through the program. Specifically, 98.8% of participants indicated that the messages were “always” or “often” positive, 96.6% reported that the messages were “always” or “often” affirming, and 93.2% rated the messages as “always” or “often” succinct (48, 52). Furthermore, 85.2% of respondents agreed that the messages were “always” or “often” relevant to their personal experiences. These findings suggest that the continuous delivery of supportive, positively framed, and relevant messaging may play a meaningful role in enhancing the mental health and well-being of participants. This aligns with previous literature demonstrating that mobile phone-based mental health interventions are often perceived as positive, affirming, and relevant by their users (48, 86, 87). Engagement with the Wellness4MDs messages was notably high. A majority of participants (93.6%) reported that they “always” or “often” read the text messages, and 73.9% stated that they not only read the messages but also took time to reflect on their content. These engagement rates are comparable to findings from previous study with similar evaluations of supportive text messaging programs (88).

Participants also expressed high levels of satisfaction with the frequency of message delivery. Approximately 84.1% of respondents indicated satisfaction with the frequency of the Wellness4MDs messages, which is consistent with previous evaluations of supportive text messaging programs (48, 88). Regarding preferences for message frequency, 60.2% of participants preferred to receive supportive messages once daily. This represents an approximate 8% decrease relative to findings from the Text4Mood program, where a higher proportion of participants preferred daily messages. In contrast, 23.9% of Wellness4MDs participants preferred receiving messages once every other day, a slightly higher proportion compared to the 12% reported in the Text4Mood evaluation (48).

Wellness4MDs, offered unique advantages over traditional and other digital mental health approaches, including scalability, accessibility, and low cost to participants compared to other interventions. Unlike in-person therapy or app-based interventions, text messaging is non-intrusive, fits easily into daily routines, and allows asynchronous access, making it particularly suitable for busy healthcare professionals. Compared to more expensive digital options like internet-based CBT (iCBT) (89), Wellness4MDs is free to users and did not require paid subscriptions or costly data plans. Additionally, it addresses critical barriers of face-to-face CBT, such as limited access and long wait times due to the shortage of trained therapists (90).

### 4.4 Implications for policy, practice and future research

The findings of this study highlight the importance of integrating digital health interventions into physician and medical learners’ wellness initiatives. Given the significant reductions in emotional exhaustion, anxiety, and well-being, future programs should continue to leverage technology-based approaches, such as mobile apps, to enhance accessibility and engagement. However, the limited improvements in depersonalization and depression suggest the need for a multi-faceted approach. Future research should explore combining individual-focused digital interventions with organizational-level changes. For example, studies have shown that reducing administrative burdens and optimizing work schedules can significantly improve physicians’ mental well-being (91–93). Implementing hybrid models that incorporate workplace modifications, peer support, and targeted mental health care may lead to more comprehensive improvements in physician well-being. Additionally, future research should examine the long-term effects beyond the three-month follow-up period. A longer study duration may offer deeper insights into sustained benefits and potential delayed improvements in depersonalization and depression. Also, we recommend incorporating qualitative assessments, such as participant interviews, that could provide valuable perspectives on the perceived benefits and limitations of these interventions. Future large-scale implementation of the Wellness4MDs SMS program could benefit from cost–benefit analyses and integration within existing healthcare systems, enabling medical institutions and organizations to ensure feasibility, scalability, and sustainability.

## 5 Limitations

Several limitations of this study should be acknowledged. First, the relatively small sample size may have reduced the statistical power of the analyses, particularly with respect to detecting significant changes in depression and depersonalization outcomes. In addition, the overall low response rate, potentially attributable to the online delivery format of the surveys, may limit the generalizability of the findings to the broader population of Wellness4MDs subscribers. Previous research has demonstrated that surveys administered via text message are associated with lower participant retention rates at follow-up compared to alternative methods, such as paper-based surveys (94, 95). Another limitation of this study is the application of the Last Observation Carried Forward (LOCF) method for handling missing data. This technique presumes that the most recent available measurement remains constant over time, which may not accurately capture actual fluctuations. Consequently, it can lead to biased findings and an underestimation of data variability. Additionally, our study did not assess the missing data mechanism or compare our handling method with alternative approaches. This will be addressed in future studies with larger sample size.

The voluntary nature of participation may have also introduced self-selection bias, as individuals with a greater interest in mental health interventions may have been more motivated to engage with the program and complete the surveys. This differential retention may have led to an overestimation of the study’s findings. Future research should consider strategies to minimize this potential bias, such as providing incentives for survey completion, simplifying survey access, or gathering brief feedback from participants who discontinue the program. Moreover, while the study employed validated and standardized self-report instruments, these measures are not diagnostic tools. Additionally, while the questionnaire used to assess user satisfaction with the Wellness4MDs program was adapted from tools applied in similar evaluations, it has not been formally validated. Also, the CBT-based SMS text messaging programs may have limited effectiveness for individuals with more complex or severe mental health conditions, such conditions often require comprehensive, individualized, and therapist-guided interventions, which cannot be fully addressed through brief, generic text-based messages alone. A further limitation relates to the operational aspects of SMS delivery. In the current study, messages were sent at fixed times and frequencies, without tailoring to individual participants’ schedules or phone usage preferences. This lack of personalization may have affected message engagement and perceived relevance for some participants. Future iteration of the program could explore adaptive scheduling or participant-driven preferences to optimize delivery and impact. It is also important to note that the effect sizes observed for significant outcomes were small, potentially limiting the practical significance of the findings. Nonetheless, this is consistent with previous research, which has shown that self-help interventions delivered without direct therapist involvement often yield small effect sizes (82, 83). Finally, the absence of a control group limits the ability to draw definitive causal inferences, as improvements observed over time cannot be conclusively attributed to the intervention alone.

## 6 Conclusion

The Wellness4MDs program offers an innovative, cost-effective approach to addressing the psychological and wellness needs of physicians and medical learners. The program significantly improved emotional exhaustion and anxiety symptoms among physicians and medical learners. Although the intervention did not lead to significant changes in depersonalization, personal accomplishment, or depression, its effectiveness in reducing burnout-related emotional exhaustion, lowering GAD-7 scores, and enhancing well-being from baseline to follow-up underscores the potential value of technology-enabled, CBT-based interventions. The Wellness4MDs program was well-received by participants, with high levels of satisfaction reported regarding the content and delivery of the supportive text messages. The study yielded important insights into participants’ self-reported experiences, suggesting that the intervention contributed to notable improvements in quality of life, overall mental well-being, and coping with symptoms of anxiety, depression, and loneliness. Furthermore, participants indicated an enhanced sense of connection to a broader support network following the intervention. However, given the small sample size, these findings should be interpreted with caution and validated in future studies with larger or more representative samples. Nonetheless, these findings provide further evidence supporting the feasibility, acceptability, and potential effectiveness of e-health interventions for mental health promotion. In particular, the positive reception among participants underscores the suitability of mobile health communication strategies for physicians and medical learners, offering a scalable and accessible approach to addressing psychological distress within this demographic.

Future initiatives should explore the long-term effects of the Wellness4MDs program beyond the three-month period and consider integrating organizational changes and targeted psychological support to further enhance physician well-being. Additionally, future randomized controlled trials are recommended to comprehensively evaluate the program’s impact on anxiety, depression, and burnout symptoms among physicians and medical learners. Given the increasing prevalence of burnout and mental health challenges in the medical profession, continued investment in digital health interventions represents a promising strategy for strengthening physicians’ psychological resilience and overall well-being.

## Data Availability

The original contributions presented in the study are included in the article/supplementary material, further inquiries can be directed to the corresponding author/s.
